# THYROID DYSFUNCTION AND THYROID ANTIBODIES IN IRANIAN PATIENTS WITH VITILIGO

**DOI:** 10.4103/0019-5154.39733

**Published:** 2008

**Authors:** Moradi Sedighe, Ghafarpoor Gholamhossein

**Affiliations:** *From Institute of Endocrine and Metabolism, Iran University of Medical Sciences, Tehran, Iran*

**Keywords:** *Thyroid antibodies*, *thyroid dysfunction*, *vitiligo*

## Abstract

Vitiligo is a common skin disorder, and the pathogenesis is unknown. An increased prevalence of autoimmune thyroid diseases has been described in these patients. The aim of this study is to assess the prevalence of thyroid dysfunction and hypoparathyroidism in patients with vitiligo.

**Materials and Methods:**

One hundred and nine patients (38 males and 71 females with vitiligo were enrolled. Thyroid physical examination was carried out. Thyroid function tests, thyroid antibodies, calcium and phosphorus were assessed. The collected data were analysed by SPSS version 11.

**Results:**

Thyromegaly was found in 30.1% of patients. Hypothyroidism was found in 16 (15.7%) out of 109 cases. Two of them had clinical and 14 had subclinical hypothyroidism. One patient had Grave's disease. Antibody positivity was the most common disorder (anti-TPO and anti-tg were positive in 36.7 and 32.1%, respectively). No patient had hypoparathyroidism.

**Conclusion:**

According to our study, thyroid dysfunction, particulary hypothyroidism and thyroid antibodies increase in patients with vitiligo. We recommend thyroid antibodies assessment and thyroid function evaluation in these patients.

## Introduction

Vitiligo is one of the most common skin disorders with a prevalence of 1-2% in different populations. The condition occurs when pigmented cells are destroyed, causing patches of skin to lose their normal color and appear whiter.^1^,^2^ The etiology of this disorder is not clear but different theories suggest that autoimmune, genetic disorders, toxic metabolites, oxidative stimuli are the main factors. The nervous system and or the absence of the melanocyte growth factor may be included.^3^

From these factors, autoimmune disorder is the most common cause and some of the patients have antibodies to melanocytes or melanocytic proteins. Although it is not confirmed that these antibodies cause disease or lead to melanocyte destruction, there is some evidence that cell-mediated immunity plays a role in melanocyte destruction.^4^–^6^ Autoimmune thyroid diseases with prevalence of up to 30% accompany vitiligo, from which hypothyroidism is one of the most common disorder.^1^

In one study of 121 children with vitiligo, 16% showed some abnormalities in the thyroid function tests, The antithyroid peroxidase antibody (Anti-tpo) was the most common disorder.^1^

In another study, the prevalence of autoimmune disorders, including thyroid disease, was more common than that of the general population.^2^

In a study, Dave and colleagues showed that the frequency of thyroid disorders (endocrine and or immunologic or both) were 57.1% in people with vitiligo in comparison to 10% in people without vitiligo. Thirty-four percent of patients in their study had thyroid antibodies.^7^

Manighalam and colleagues conducted a study on 30 patients with vitiligo and they found hyperthyroidism and hypothyroidism in 10 and 6.6%, respectively.^8^ In another recent report from Iran, antithyroid antibody was detected in 18.1% of patients with vitiligo in comparison to 7.3% in control group with more prevalence in females.^9^ The aim of this study is to investigates the percentage of thyroid dysfunction and antithyroid antibodies in Iranian patients with vitiligo.

## Materials and Methods

This is a cross-sectional study that was conducted between 2005 and 2006 in 109 patients with vitiligo (38 males and 71 females) with a mean age of 34.4 ± 13 years.

A dermatologist diagnosed the condition as vitiligo at least 6 months before the initiation of the study. All the patients underwent thyroidal physical examination for the presence of goiter.

Laboratory tests included the following: Thyroid stimulating hormone (TSH) measured by IRMA (pars kit normal limits: 0.3-3.5 mIU/ml)

T3, T4, and T3RU were measured with RIA method (pars-kit). The anti-TPO and anti thyroglobulin antibody (Anti-Tg) were measured using the RIA methods with the normal range being below 30.

Serum calcium and phosphorus were measured in all patients, and if the results were abnormal, the measurement was repeated and parathyroid hormone (PTH) was measured as well.

### Statistical analysis

Student's *t*-test/chi-square/and Mann-Whitney U tests were performed. All the data was analysed with SPSS, version 11.

## Results

One hundred and nine patients were enrolled. The mean age was 34.41 ± 13 (CI: 8-65) years [71 females (65%) and 38 males (35%)]. Seventy-five patients had a normal thyroid size and 34 patients (30.1%) had goiter.

The laboratory findings are shown in Table 1.

**Table 1:** Laboratory findings in patients with vitiligo

	Mean	SD	Minimum	Maximum	Range
TSH (mIU/L)	2.15	±5	0.2	50	0.3-3.5
Anti-Tg (IU/ml)	212	±482	1.2	3074	<30
Anti-Tpo (IU/ml)	248	±649	2.4	3427	<30
Ca++ (mg/dl)	9.1	±0.5	8.1	10.15	8.5-10.5
P (mg/dl)	3.1	±0.57	2.3	5.19	2.5-5.5

Nineteen patients (17.4%) had abnormal TSH levels and in three patients (2.8%), the TSH level was less than normal, one patient had Grave's disease and in another two patients, T3 and T4 were normal.

In 16 patients (15.7%), TSH levels were more than normal, and in 12 of them, it was more than 5 mIU/l. In these 16 cases, there were 12 females and 4 males. Two patients had clinical hypothyroidism. Anti-Tpo and anti-Tg antibody were positive in 40 (36.7%) and 35 (32.1%) cases, respectively. Thirty-four patients had goiter, from which 14 of them (42.4%) had abnormal TSH levels and this correlation was significant with Mann-Whitney U test (*P* = 0.002) and 21 of them (67.7%) had anti-Tpo antibody (*P* = 0.001).

There was a positive correlation between TSH and anti-Tpo antibody (*r* = 0.4, *P* = 0.002).

One case had serum calcium levels less than normal and two had increased serum phosphate levels, which was normal after tests was repeated and PTH was normal in all cases. Therefore, no patient had hypoparathyroidism.

## Discussion

Vitiligo is a common skin disorder in which skin depigmentation is due to destruction of melanocytes and decreased melanin. Although the exact pathogenic processes involved in the destruction of melanocytes in vitiligo are still unknown, autoimmune melanocytic destruction have been advocated.^3^ This disorder always accompanies other autoimmune diseases, and the presence of autoantibodies is used to prove this theory.^10^

We carried out this study in order to determine the association between autoimmune thyroid diseases, parathyroid disorder with vitiligo. Nineteen (17.4%) patients had overt thyroid disorders with hypothyroidism as the most common presentation and one patient had Grave's disease. These results can be compared to those of other studies.

In a study that was performed on 121 children with vitiligo (the group that has less thyroid disorders), nine patients had hypothyroidism and one patient had hyperthyroidism.^1^ In another study that was conducted in India, the thyroidal disorders were more common and in 35 patients with vitiligo, 40% had thyroidal dysfunction and 34.1% were anti-tpo antibody positive.^7^ In a research that was carried out on 30 patients with vitiligo in Iran, hyperthyroidism and hypothyroidism was observed in 10 and 6.6% cases, respectively.^8^ In a recent report from Iran, there was no thyroidal dysfunction, but the anti-tpo antibody positivity was more than that of the control group.^9^

The important findings of our study were the increased positivity of anti-tpo and anti-tg antibodies.

Forty patients (36.7%) and 32.1% cases had elevated anti-tpo antibody and anti-tg antibody, respectively.

In other studies, the positive anti-tpo antibody and Anti-tg antibody were the most common disorders. In one study, the positive anti-tpo antibody was observed in 10% children; however, this can be due to the lower age group of this study, in which the children normally have lower antibody level than that of the adults.

In a study by Daneshpazhooh and colleagues, increased anti-tpo antibody levels were found in approximately 18% of their patients.^9^ Although the frequency of positivity of anti-tpo antibody in their study was different to our findings, it could be due to the age group of their study sample and also female was more predominant in our study.

In study that was carried out in India, the anti-Tpo antibody was positive in 31.4% cases, which was similar to our study.^7^ In a study that was conducted in UK in 40 patients, 34% had positive antithyroid antibody,^11^ and in a report from Australia, 106 patients 21% had positive antithyroid antibody.^12^ In Greece, in 54 children and adult cases with vitiligo, the prevalence of anti-tpo antibody was 24.1%.^13^

In our study, 34 patients (301%) had some degree of goiter and most of them were women. Further, in another study that was performed in Iran, the prevalence of goiter was 20%.^9^

This may be due to high number of women in our study, who normally have more thyroid enlargement than men.

There are few studies that search the relation between vitiligo and hypoparathyroidism.

In a study by Betterle and colleagues, the prevalence of autoimmune parathyroid disease in patients with vitiligo was 1%.^14^ We found no abnormalities in serum calcium, phosphorus and PTH levels; therefore, we did not detect any patients with hypoparathyroidism.

In conclusion, our study showed that the autoimmune thyroid diseases, most commonly hypothyroidism and autoimmune thyroiditis that are confirmed with the presence of antithyroid antibody, are more common in patients with vitiligo than general population.^15^

We recommend the measurement of the TSH levels and anti-Tpo antibodies in all the patients with vitiligo and all of them who have a high level of anti-Tpo antibodies should be followed-up annually with TSH. It should be measured every several years in all patients because thyroid dysfunction can exacerbate general signs and symptoms such as depression or a degree of low quality of life in patients along with chronic skin disorders.
